# Temporal, biomechanical evaluation of a novel, transcatheter polymeric aortic valve in ovine aortic banding model

**DOI:** 10.3389/fcvm.2022.977006

**Published:** 2022-12-20

**Authors:** Mateusz Kachel, Piotr P. Buszman, Krzysztof P. Milewski, Magdalena Michalak, Wojciech Domaradzki, Maciej Pruski, Michał Sobota, Carlos Fernandez, Marta Konopko, Jerzy Nożyński, Paweł Kaźmierczak, Jakub Włodarczyk, Mateusz Stojko, Andrzej Bochenek, Paweł E. Buszman

**Affiliations:** ^1^American Heart of Poland, Center for Cardiovascular Research and Development, Katowice, Poland; ^2^Department of Cardiology, Andrzej Frycz Modrzewski Kraków University, Bielsko-Biała, Poland; ^3^Faculty of Medicine, University of Technology, Katowice, Poland; ^4^Department of Epidemiology, Medical University of Silesia, Katowice, Poland; ^5^Centre of Polymer and Carbon Materials, Polish Academy of Sciences, Zabrze, Poland; ^6^Silesian Center for Heart Diseases, Zabrze, Poland

**Keywords:** aortic valve stenosis, transcatheter aortic valve replacement (TAVR), heart valve prosthesis, polymeric valve, preclinical “*in vivo*” study

## Abstract

**Objectives:**

The aim of the study is to evaluate the functionality, durability, and temporal biocompatibility of a novel, balloon-expandable polymeric transcatheter heart valve (ATHV) system (InFlow, CardValve Consortium, Poland). Along with expanding TAVI indications, the demand for new transcatheter valves is increasing.

**Methods:**

A surgical ascending aortic banding model was created in 20 sheep. Two weeks later, 16 sheep were implanted with ATHV systems (15–16F). Three animals were euthanized after a 30-day follow-up, four animals after a 90-day follow-up, and six animals after a 180-day follow-up. A follow-up transthoracic echocardiography (TTE) was performed.

**Results:**

There was one procedure-related (6,25%) and two model-related deaths (12,5%; banding site calcification with subsequent infection originating externally from banding). TTE revealed the flow gradients (max/average) of 30,75/17,91; 32,57/19,21; and 21,34/10,63 mmHg at 30, 90, and 180 days, respectively. There were two cases of low-degree regurgitation after 180 days with no perivalvular leak observed. Histopathological analysis showed no valve degeneration at terminal follow-up with optimal healing. Small thrombi were present at the aortic wall adjacent to the base of the leaflets, and between the aortic wall and the stent in most of the valves; however, leaflets remained free from thrombi in all cases. Scanty calcifications of leaflets were reported in three animals evaluated 180 days after implantation.

**Conclusion:**

This preclinical study in the aortic banding model showed good hemodynamic performance, durability, and biocompatibility of the novel ATHV. Furthermore, regulatory studies with longer follow-ups are warranted.

## Introduction

Transcatheter aortic valve implantation (TAVI) marked a beginning of a new era in aortic valve disease treatment (1). TAVI has come a long way from the last call technique for inoperable patients, through an equal measure in high-risk patients, to recent expansion of indications, becoming a favorable option in elderly patients and confirming its non-inferiority in moderate and low-risk patient subsets (2, 3). However, of note, the long-term results in these groups are still missing. This new reality creates more challenges for new TAVI technologies and increases the pressure to further improve outcomes and overcome current limitations of TAVI, including paravalvular leaks, vascular complications, and long-term durability by introducing novel solutions and upgrades to currently available technologies. However, the main limitation, the cost of TAVI combined with its accessibility, is yet to be answered. Currently, approximately 180,000 patients can be considered potential TAVI candidates in the European Union and Northern America annually. Even highly developed and wealthy countries struggle to cover the need. This situation might be further aggravated, as the potential future expansion of indications to lower-risk groups is expected to increase demand for transcatheter valves up to 270 000 (4).

Nowadays, long-term evaluation in the preclinical setting of new THV technologies has been limited to acute feasibility studies and very limited to long-term evaluation due to a lack of calcifications and anchoring mechanisms in the healthy animal aortic valve. The only available model of THV implantation in descending aorta with the creation of aortic valve insufficiency was related to high mortality (5).

Most recently, we introduced a novel ovine model of aortic banding and TAVI, which allows for stable valve anchoring and long-term evaluation, including mechanical performance and biological response (6).

Bearing in mind the above-mentioned limitations, an innovative polymeric ATHV would outrun the biological counterpart in terms of durability and become more affordable and thus attainable. Herein, we present the results of a short-, mid-, and long-term evaluation of mechanical performance, durability, and biological response of a low-profile, polymeric THV (InFlow, CardValve consortium) based on the novel ovine model of aortic banding.

## Materials and methods

### Study design

The study protocol has been accepted by the local ethics committee for animal research, Decision No. 150/2016. All animals received the standard of care outlined in the study protocol and in accordance with the act of animal welfare and the “Guide for the Care and Use of Laboratory Animals” (7). Slight ascending aorta stenosis (AS) was created by fixing a surgical band around the aorta. After AS creation, animals were allowed a recovery period of at least 10 days. Subsequently, an InFlow ™ transcatheter heart valve was implanted *via* TAVI techniques and a carotid artery approach. Follow-up echocardiography and complete blood works were performed at 30-, 90-, and 180-day follow-ups. Twenty blackface crossbreed sheep, approximately 2 years old, weighing 40 to 80 kg were included. Animals received an acclimation period of at least 21 days.

### Study device

**FIGURE 1:**
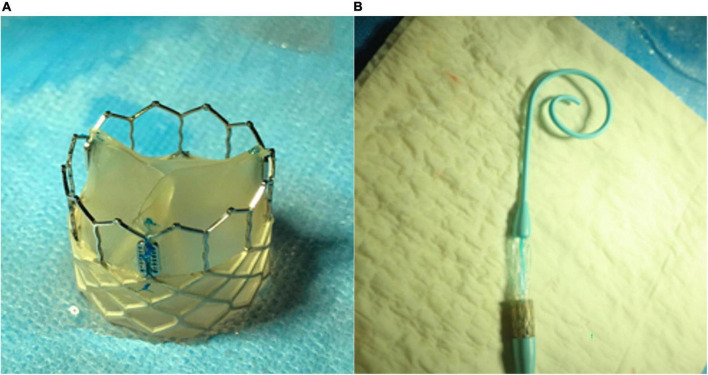
**(A)** Polymeric InFlow artificial heart valve prototype—lateral view and **(B)** InFlow valve crimped on the balloon.

**FIGURE 2:**
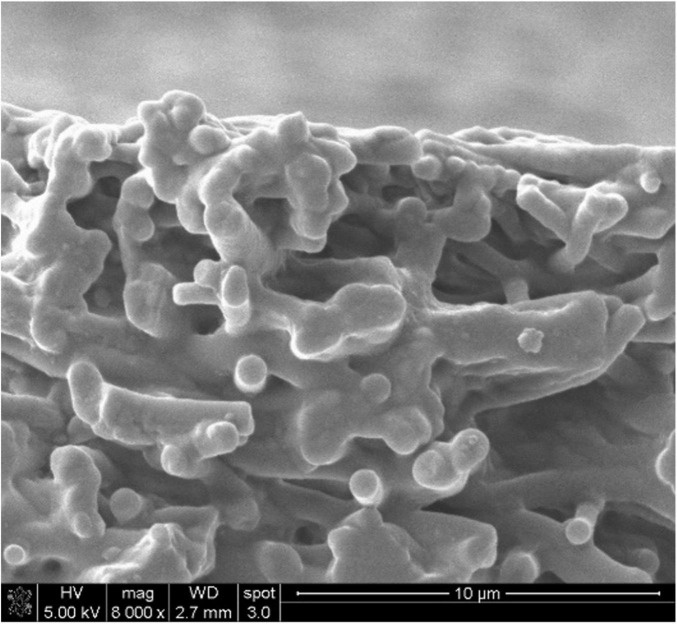
A cross-section of a single leaflet was imaged using scanning electron microscopy (SEM) (mag. 8000x).

An InFlow™ Artificial Transcatheter Heart Valve (ATHV) comprises a proprietary balloon-expandable, radiopaque, cobalt–chrome alloy frame, and a tri-leaflet polymeric valve connected with a cuff, made from a combination of different polymers (Figures 1, 2). The copolymers of ChronoFlex Ar 22% (polyurethane-co-carbonate) (PU) and ChronoSil AL80A 5% (polycarbonate-co-silicone) (PUS) manufactured by AdvanSource (Wilmington, MA, USA) were used for heart valve leaflets preparation. Using the electrospinning unit model NEW-BM (NaBond), these polymers were processed to the multilayer fibrous and semi-fibrous layers mounted on stents. Polymeric materials are attached to the metal frame using the electrospinning method enabling the limited use of standard suturing and thus reducing the possible damage done to the material and plausible subsequent complications. The ATHV is a terminally sterilized (radiation), single-use device, indicated for relief of AS in patients with symptomatic heart diseases due to severe native calcified AS in patients at high or greater risk for open surgical valve replacement. For the study purpose, the InFlow™ transcatheter heart valve was available in diameter equaling 23 mm and used with a dedicated delivery system including a pig-tail catheter and a dog-bone-shaped balloon for better control and landing accuracy. After proper crimping, the outer diameter of the device is 15–16F. Devices are stored in a glutaraldehyde solution. This transcatheter artificial heart valve and delivery system are covered with five international patents issued (no. P.426429, P.426432, P.426433, P.426434, and P.426463).

The study device was previously subjected to bench testing. Six artificial heart valves were tested using BDC Laboratories VDT-3600i pump, dedicated equipment for the durability test. According to ISO 5840, the temperature of the test was 36, 6°C. Waveform, frequency, and stroke conditions (4,5 ml–10,46 ml) were all adjusted in the same manner for all tested prototypes. To meet preclinical study purposes and 6-month follow-up, all valves passed 40 million cycles. Further tests are ongoing and warranted.

### Aortic banding model

Sheep were anesthetized using a combination of ketamine 10 mg/kg IM/IV + xylazine 0.05–0.2 mg/kg IM + atropine 0.1–0.2 mg/kg IM. Propofol 2–4 mg/kg IV was administered to facilitate intubation. Following successful intubation, sheep were placed in the right lateral recumbency. Aortic banding was achieved by means of a minimally invasive left-side thoracotomy. An incision was made between the fourth and fifth intercostal space, and the ascending aorta was exposed. The target site for the banding implantation was mid-way from the native aortic valve and the common carotid trunk. With the help of sizer kits, the Dacron sleeve was measured, and the diameter of the aorta decreased between 2 and 4 mm. A surgical stainless-steel wire was sutured in the mid-line of the banding tissue to allow identification under fluoroscopy. After the procedure completion, the wound was closed, and the sheep moved to post-op recovery.

### Transcatheter aortic valve implantation

Two weeks after the aortic bending, TAVI procedures were performed starting with anesthesia using the same procedures outlined for the banding. Sheep were placed in dorsal recumbency with the legs stretched caudally. The left carotid artery was surgically exposed and prepared, as close to the thoracic inlet as possible. A 6fr arterial sheath was placed in the carotid artery. A J wire 0,035” was advanced through the arterial sheath in the left ventricle, and a 5fr pig-tail catheter with 10 mm markers was advanced over the J wire. Ventriculography and aortography along with invasive pressure evaluation were performed to assess the banding site and measure the target implantation site diameter. The pig-tail catheter markers were used to calibrate the distance. After all the measurements were finished, the pig-tail catheter and the J wire were removed. The ATHV valve was crimped on a balloon matching the valve size (a 23-mm balloon for a 23-mm valve) and the natural direction of blood flow from the heart (aortic position). The 6fr arterial sheath was removed and replaced with an arterial sheath bigger than the measured profile of the valve (usually 18–22F). Once the large arterial sheath was inserted, heparin was administered at a dose of 300 IU/kg (3 mg/kg), IV, to achieve an activated clotting time (ACT) over 300 s. A super stiff Amplatz wire was advanced through the arterial sheath into the left ventricle. The valve crimped on the balloon was advanced over the Amplatz wire and through the arterial sheath to the aortic banding. The valve was expanded with the help of a 50-ml syringe filled with a 70:30 ratio of saline and contrast. Once the implantation was complete, the Amplatz wire and the balloon were removed. Post-implantation control ventriculography and aortography were performed as outlined earlier without changing the arterial sheath. The arterial sheath was removed, the carotid artery ligated, and the tissues and skin sutured in three layers. The sheep were then transferred to post-op recovery.

### Echocardiography

Transthoracic echocardiography (TTE) was performed at 30, 90, and 180-day follow-ups. Transoesophageal echocardiography (TEE) was performed at 180 days as a complement to TTE, while under anesthesia. All routine parameters were evaluated (left ventricle end-diastolic volume, aortic diameter, left ventricle end-systolic diameter, ejection fraction, cuspids’ separation, among others), and valve functionality, deployment, and any other visual findings were documented in the echo reports.

### Pathological evaluation

The independent, pathology core lab (Silesian Centre for Heart Diseases, Poland) received fixed, explanted hearts and ascending aorta for histopathology. Hearts were trimmed and the segment of tissue containing the explants was excised, grossly examined, and radiographed. Aortic roots with valve implants (AV) were dehydrated in a graded series of ethanol, cleared in xylene, and infiltrated and embedded in SPURR plastic resin. After polymerization, the device with the frame was sectioned radially two times to capture each cusp (LCC = left coronary cusp; RCC = right coronary cusp; NCC = non-coronary cusp) and stained with hematoxylin and eosin (H&E). In addition, the portion of the plastic block containing each of the three valve cusps (radial planes) was separated from the frame, cut serially two times (thin sections), and stained with Movat’s pentachrome (MP) and Von Kossa (VK). The block remnants were reassembled with appropriate spacers and cut crosswise (transverse plane) at two levels. All ground sections were ground and micro-polished to an optical finish using the Exakt cutting/grinding system. Resulting sections were stained with H&E. Trackable gross lesions submitted separately were processed, embedded in paraffin or SPURR resin as appropriate, sectioned, and stained with H&E and/or Masson’s trichrome (MT) (paraffin only). All resulting slides were evaluated via light microscopy by the study pathologist. In the event of identifying problems with valve function, the harvested tissues were passed to the histopathology analysis. If no correlation between reported death and valve function was revealed, further analysis was abandoned.

### Statistics

This is a prospective, observational, and experimental study; therefore, no study hypothesis was made. Data are presented as medians (25th–75th percentile). To test for temporal differences in echocardiographic parameters, a repeated measures ANOVA has been performed followed by a pairwise comparison with the Bonferroni modified paired *t*-test. A *p*-value of <0.05 was considered statistically significant. MedCalc Statistical Software version 14.12.0 (MedCalc Software bvba, Ostend, Belgium^1^) was used for analysis.

## Results

**FIGURE 3:**
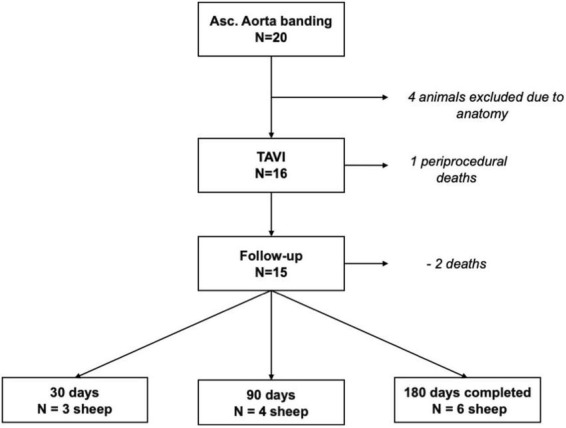
Study flowchart.

The study flowchart is shown in Figure 3. All 20 sheep survived the banding procedure, from which 16 were preselected according to banding location and size for the testing of ATHV. There was one procedure-related death within 7 days after TAVR. Three designated animals were euthanized after a 30-day follow-up, four animals after a 90-day follow-up, and six animals after a 180-day follow-up. There were additional two deaths during the follow-up (detailed description later). The detailed cause explanation is shown in Table 1.

**TABLE 1:** Causes of premature death.

Cause of death	Number of animals
Death in post-operational care (inability to resume respiratory function after anesthesia) – day 0	1
Calcification and vegetation in banding site – day 18 and 62	2

### Echocardiographic results

**FIGURE 4:**
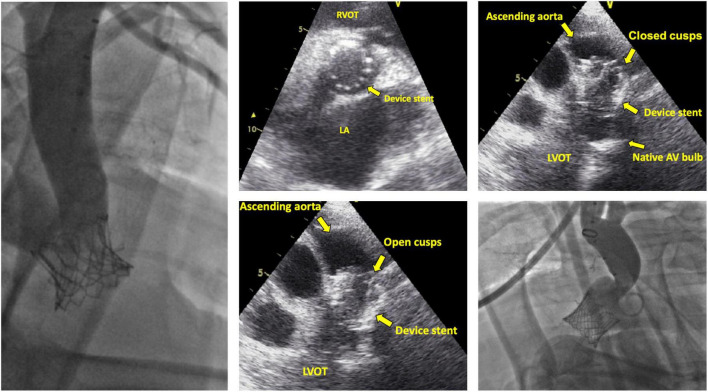
Fluoroscopy and echocardiography images of the implanted prosthesis.

**FIGURE 5:**
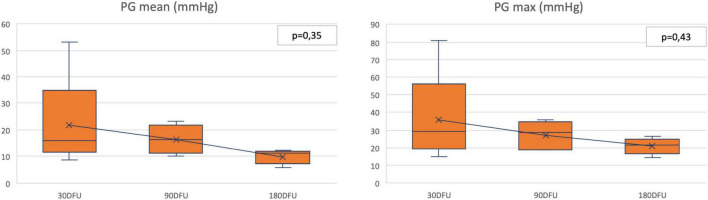
Pressure gradients—serial measurements. DFU: days follow-up; PG: pressure gradient.

Echocardiographic analyses were conducted according to the protocol at respective time points of 30, 90, and 180 days. Representative images are shown in Figure 4. At the time of terminal control, TTE was utilized complementary to the standard transthoracic echo after the previous induction of anesthesia (at the time point of 30 and 90 days, only transthoracic echo was performed). Examination showed good hemodynamic results for all the valves at respective time points. Maximal and mean transvalvular gradients were typical for percutaneously implanted valves indicating proper valve deployment. At follow-up in TTE, the maximum and average flow gradients [median (IQR) – presented as max/average] for 30-, 90-, and 180-day observation were 26,4(18,9–34,7)/14(12,1–20,1); 30,7(23–35,5)/16,6(13,9–22,4); and 22,2(19,3–23,7)/11,4(9,3–12,1) mmHg, respectively. Serial measurements performed in five animals (one was disqualified because of a lack of available results at 30 days due to difficult examination conditions) showed that the valve hemodynamic was stable through the whole observation period (Figure 5). Valvular regurgitation was rare with no episodes of severe valvular regurgitation; however, one case of moderate-grade valvular regurgitation was reported. No perivalvular leaks were reported. Detailed ECHO parameters are shown in Table 2.

**TABLE 2:** Echocardiography findings.

	30 DFU		90 DFU		180 DFU	
Doppler measurements	Median	Q1–Q3	Median	Q1–Q3	Median	Q1–Q3
V max (m/s)	2,6	2,2–3,0	2,8	2,4–3,0	2,4	2,2–2,4
PG max (mmHg)	26,4	18,9–34,7	30,7	23,0–35,5	22,2	19,3–23,7
PG mean (mmHg)	14,0	12,1–20,1	16,6	13,9–22,4	11,4	9,3–12,1

**ECHO findings**	***n*** **= 15**	**%**	***n*** **= 10**	**%**	***n*** **= 6**	**%**

Mild regurgitation	2	13,33	2	20	2	33,33
Moderate regurgitation	1	6,66	1	10	0	0
Possible calcification	2	13,33	1	10	1	16,6
Present calcification	2	13,33	0	0	1	16,6
Probable vegetation	0	0	0	0	1	16,6
Mean pressure gradient > 30 mmHg	1	7,14	1	10,0	0	0

### Histopathology results

**FIGURE 6:**
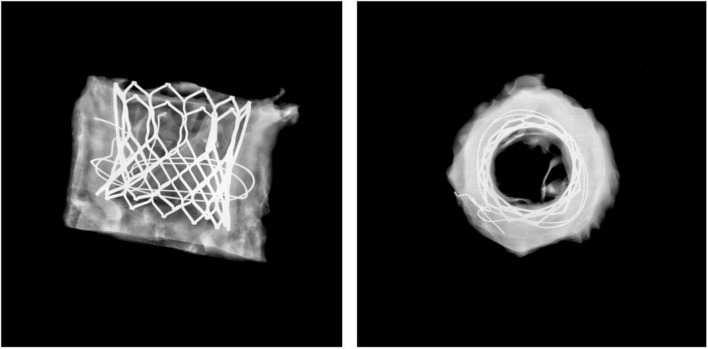
X-ray image. No deformations or damages in stent geometry.

Histopathological analyses showed good positioning of the valve in all cases. In each case, X-ray studies at lateral and craniocaudal projections showed a lack of stent deformations (Figure 6).

In the 30 days group (three cases), the gross inspection showed elastic leaflets, without tears, fenestrations, or other pathologies. Metallic stent elements and the base of the leaflets were covered by a thin layer of neointima. The leaflets were free of thrombi. Only between the base of the leaflets and the aortic wall, a thin layer of clots was formed. X-ray analysis showed no calcifications of leaflets in all cases, free margins, and commissures. Pannus was well recognized at the lower part of the implanted prosthesis as the immature tissue with an abundance of extracellular matrix and hemosiderin deposits.

Similarly, in the 90-day group (four cases), the analysis showed thin elastic leaflets with no tears, fenestrations, or focal thickenings. A thin thrombus was visible on the ventricular surface in three animals, presenting as surface deposits, well delineated, and firmly attached to the base of the leaflets and adjacent stent elements contacting the aortic wall. X-ray observations indicated only one case of focal punctiform calcifications in place of commissures. Generally, stent struts were covered by immature and early matured neointima at above 75% of their surface. Sutures were also covered partially with neointima. In all cases, the absence of inflammatory infiltrations was obvious. Translucent leaflets showed no other foreign elements. It should be pointed out that the penetration of cellular elements into the polymer was never reported. Fibrous pannus was present only at the bases of leaflets. Histology of leaflets showed linear and focal cellular covering in three cases. In one case, thin fibrin deposits were present.

**FIGURE 7:**
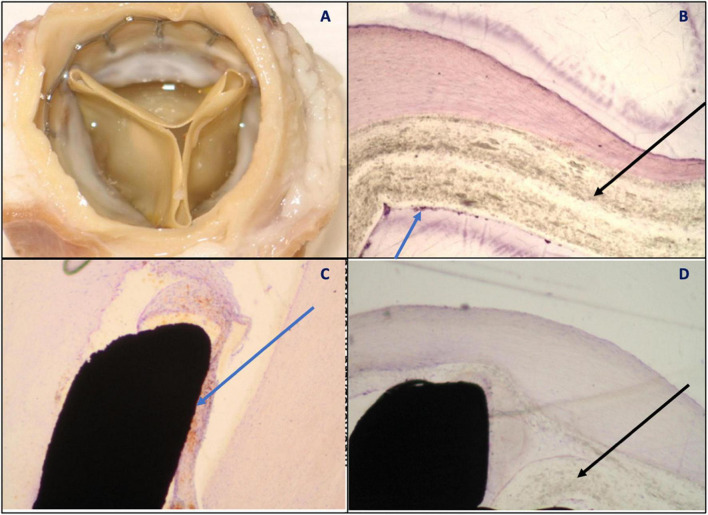
**(A)** Vascular surface—thin cusps, unchanged, metal frame covered with relatively thick neointimal tissue indicating pannus formation. No thrombus visible; **(B)** polymer (black arrow) partially covered with neointima (blue arrow) encompassing single cells; **(C)** stent-tissue contact zone (blue arrow) with no signs of inflammation; and **(D)** polymer (black arrow) with no signs of resorption or penetration of cellular elements into the polymer.

The 180-day group consisted of six cases. In one case, there was firm vegetation with outflow stenosis, but no thrombi. Small thrombi were found at the aortic wall adjacent to the base of the leaflets and between the aortic wall and the stent in two cases. The leaflets were free from thrombi. The surface of the leaflets was elastic and smooth, without tears or fenestrations. Neointima was present covering only the stent and the sutures (leaflets were left uncovered). Punctiform calcifications of leaflets and commissures were present in three cases subjected to the analysis. Histopathology of leaflets showed a translucent structure of the polymer, focally with cells adjacent to the leaflet surface (Figure 7). No inflammation inside the polymer structure was reported.

The analysis of two animals that died prematurely on days 18 and 62 has shown banding site calcification with subsequent infection originating externally from the aortic banding. A discussion of this is provided below.

## Discussion

The preclinical assessment of the InFlow polymeric THV demonstrated the feasibility of implantation, functionality, durability, and biocompatibility of a novel prosthesis both in short- and mid-term observation in an ovine model of aortic banding and THV implantation. The utilization of this innovative model resulted in a repeatable anchoring process that allowed for the successful implantation of the tested valves as well as temporal evaluation for up to 6 months. The mortality rate at follow-up was minor, and the reasons for death were not device-related, but procedure- or model-related. At terminal follow-up, histopathological analyses confirmed good positioning of the prostheses in all cases, good biocompatibility, and with early endothelialization.

The echocardiographic evaluation showed good hemodynamic results in respective time points with transvalvular gradients and velocities within normal limits. This is significant, given the fact that, as stated in the histopathology report, the presence of neointimal pannus covering the stent surface and leaflets to varying degrees (as seen in the 30- and 90-days follow-up) could have also influenced the gradient by inducing relative vessel stenosis and slightly impairing leaflet mobility. The pannus tissue showed maturation increased with time but in all cases, the pannus never formed a stenotic “collar” visible in implanted orthotopically valves. This phenomenon was reported in other studies of the biological valve utilizing surgical aortic banding and thus is considered model-related (8). Importantly, no severe cases of prosthesis insufficiency were reported, with only two cases of moderate regurgitation. Good anchoring capabilities of the landing zone and sustainable radial force generated by the metal stent frame resulted in proper sealing, and no perivalvular leakage was observed throughout the study. In addition, the innovative polymeric material was developed and used to create the leaflets, and the cuff of the prosthesis was proved to be biocompatible and non-thrombogenic with neointima covering the majority of the surface of the stent from 90 days on as seen in the pathology.

In our study, we reported two animals that died during the observation period. Every single case was thoroughly evaluated in search of a possible explanation and any potential prosthesis malfunction that could influence the outcome. The above-mentioned animals were found dead after 18 and 62 days, respectively. The post-mortem analysis unveiled that in both cases the possible cause was a heavy calcification of the banding region with subsequent infection and vegetation on the prosthesis that immobilized the valve and resulted in heavy cardiac insufficiency (one of the animals was found with hydrothorax). Interestingly, as seen in the histopathology, the calcification process originated externally from the prosthesis and then penetrated the valve itself. Presumably, this can be attributed to the potential infection of the banding site that developed after the initial surgical procedure and turned into a chronic inflammatory process that resulted in calcium deposition, hindered hemodynamics, and vegetation that led to valve failure. Independent pathological analysis qualified this event as banding model-related. Our reports of harvested and analyzed bandings without TAVI implantation show that calcification and osseous metaplastic processes are occurring in this region, supporting our above rationale. Apart from these, no other adverse events were reported.

The above-described mortality rate of the polymeric InFlow valve equaled 6,25% in the periprocedural period (1 out of 16) and 13,3% in the observation period (2 out of 15), respectively. The mortality is similar to our most recent study utilizing biological THV in the banding model (8). However, these numbers are significantly lower when compared to previous studies of THV technologies utilizing either the descending aorta model or implantation in the native position (5, 9, 10). This is mostly due to improvements in the animal model design, which resulted in improved valve anchoring, no valve dislocation, and, as a result, improved survival.

Another important aspect is valve durability and biological response. Structural valve deterioration (SVD) is a gradual process defined by permanent intrinsic changes in the valve (calcification, pannus, and leaflet failure) leading to degeneration and/or dysfunction, which in turn may result in valvular stenosis or intra-prosthetic regurgitation (11). Although SVD is well documented in surgical valves (12, 13), TAVI studies such as PARTNER 1 with 5-year follow-up failed to demonstrate the importance of this phenomenon (around 0,2% deteriorated prostheses requiring management) (14). Despite reassuring results in the midterm, the lack of longer observations available constitutes a serious limitation as SVD events were hardly reported in transcatheter heart valves in the first 10 years after the initial procedure due to insufficient follow-up (15). Therefore, obtaining convincing data about the durability of current THV already at the experimental stage is of high importance. At long-term follow-up in the current preclinical study of the InFlow ATHV, there were two of 15 (13,3%) degenerated valves, but in pathology, the calcifications originated from the banding and were qualified as model-related. In a study in which no THV was implanted, the banding site itself created calcification and ossification originating from the graft (Supplementary Figure 1) (6). Therefore, to the best of our knowledge, no device-related SVDs were identified in this study. The evidence from the preclinical studies of currently available THV is very limited. The currently evaluated ATHV InFlow system shows similar healing with no degeneration at the comparable period to biological balloon-expandable THV counterparts (5, 8–10).

### Limitations

The presented study includes several limitations that have to be considered: First, although the aortic model banding model was created, the included animals were young and healthy, with no calcific native valve stenosis.

Finally, as mentioned in the methodology section, the prostheses were implanted in the ascending aorta region, pre-prepared with the banding procedure. Such a scenario did not require the removal of the native aortic apparatus, and thus a potential bias attributed to the proper function of a native valve and different hemodynamics could be perceived.

## Conclusion

The study showed a proper hemodynamic performance and acceptable biocompatibility of the novel artificial polymeric InFlow ATHV, similar to biological counterparts, as evaluated in the same follow-up in the ovine banding model. Given the presence of micro-calcifications and microthrombi on several valves, a finding was also reported in other preclinical studies including biological valves (8). Further studies with longer follow-ups are warranted. The presented prosthesis may be a viable alternative to the currently used biological technologies and add up to the widespread utilization of TAVR procedures and long-term durability.

## Data availability statement

The raw data supporting the conclusions of this article will be made available by the authors, without undue reservation.

## Ethics statement

The animal study was reviewed and approved by Local Ethics Comittee in Katowice.

## Author contributions

KM, AB, PK, MM, and PEB were responsible for planning the whole study. WD, MP, CF, and MKo were responsible for conducting procedures of creating aortic banding model and transcatheter aortic valve implantation. MS and JN were liable for reporting the work described in the article. MKa and PPB were being responsible for the overall content as guarantors and they took part in planning, conducting, and reporting results of the study. All authors contributed to the article and approved the submitted version.

## Footnotes

1
http://www.medcalc.org

## Supplementary material

Supplementary Figure 1 **(A)** Visible growths from the heart or vessel wall, extending over the stent, including the cusps and narrowing the opening. **(B)** Visible calcifications with formation of concretions near the edges of free cusps. **(C)** Radiological magnitude of the concretions reveals a trabecular fibrous structure presumably associated with the tissues of the annulus.
