# Supplementary figures and images for: Temporal, biomechanical evaluation of a novel, transcatheter polymeric aortic valve in ovine aortic banding model
Source: Front Cardiovasc Med. 2022 Dec 20;9:977006. doi: 10.3389/fcvm.2022.977006 (PMC9810075; doi:10.3389/fcvm.2022.977006)

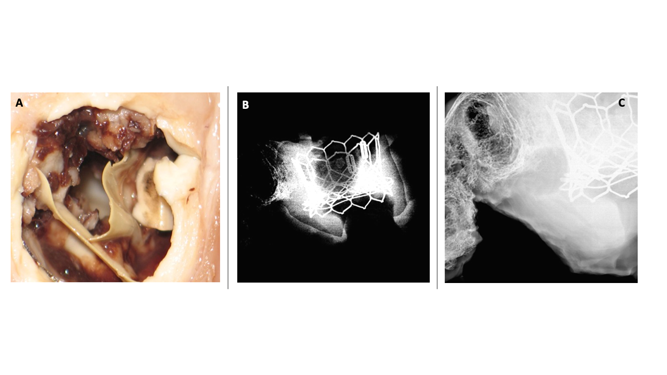

Supplement: Supplementary Figure 1 — (A) Visible growths from the heart or vessel wall, extending over the stent, including the cusps and narrowing the opening. (B) Visible calcifications with formation of concretions near the edges of free cusps. (C) Radiological magnitude of the concretions reveals a trabecular fibrous structure presumably associated with the tissues of the annulus. [file Image_1.TIFF]
